# Patricia Gallagher (1954–2021)

**DOI:** 10.5195/jmla.2022.1478

**Published:** 2022-04-01

**Authors:** Stephen J. Greenberg

**Affiliations:** 1 patzere4@gmail.com, Retired

Patricia Gallagher, MA, MLS, AHIP, FMLA, passed away on December 1, 2021, after a ten-month battle with pancreatic cancer. She was sixty-seven years old.

To those who knew her best, Pat was always the quintessential New Yorker. She was born in the old Metropolitan Hospital on Welfare (now Roosevelt) Island in the East River, where her mother worked as a nurse, and her father managed the hospital telephone system. Her parents took her home to their apartment on 22^nd^ Street, between First and Second Avenues in Manhattan, and she lived on that city block for over fifty years.

Pat attended Catholic schools in New York City, including Cardinal Spellman High School, where a classmate was Supreme Court Justice Sonia Sotomayor. After completing her BA (cum laude) at Lehman College and her MLS at Queens College, she entered medical librarianship in 1978 at the Helene Fuld School of Nursing Learning Center. In 1987, Pat took a position at the Seymour Philips Library at the Beth Israel Medical Center (walking distance from her apartment). It was at Beth Israel that Pat became active in the Medical Library Association (MLA), both with her local chapter and nationally. She began producing a long series of articles, presentations, continuing education (CE) classes, and websites touching many areas, from urban health to the history of the health sciences. She joined the Academy of Health Information Professionals in 1989 and reached distinguished status in 1994, which she maintained for the rest of her life. She cowrote two editions of the MLA BibKit on the History of the Health Sciences and almost single-handedly maintained the History of the Health Sciences section's web-links page for over two decades. Between 1987 and 2021, she did not miss an MLA annual conference.

In 1995, she left Beth Israel to work at the New York Academy of Medicine (NYAM). It was at NYAM that she became involved with NOAH, “New York Online Access to Health,” a groundbreaking public health resource database that she would eventually lead as managing editor. Among other distinctions, NOAH was a pioneer in offering parallel English and Spanish content. NOAH was awarded the MLA Frank Bradway Rogers Award in 2006 for “distinguished professional contributions to the application of technology in the delivery of health care information.” Accepting the award on behalf of the contributors and editors was one of her proudest professional moments.

**Figure:**
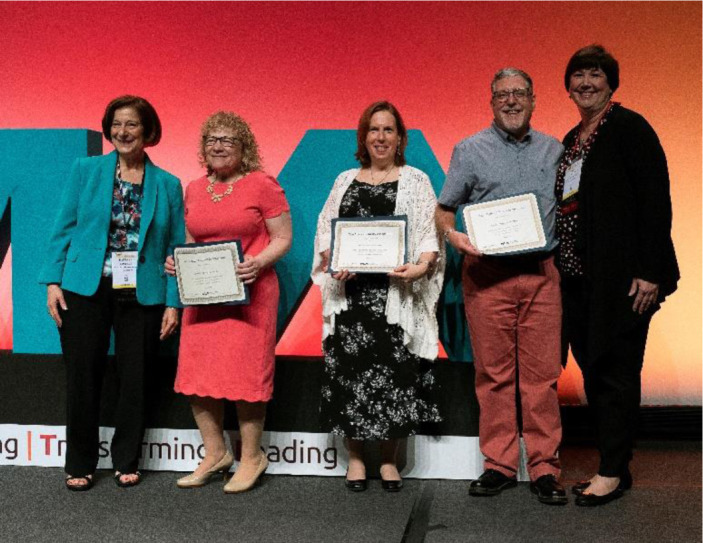
Pat becomes an MLA Fellow, 2018. Left to right: Barbara Epstein, Elaine Martin, Pat, Jerry Perry, Teresa Knott.

Her activities at NYAM were not limited to NOAH. Starting in 1998, she worked with Drs. Peter Wyer and Barney Eskin to coordinate a national course, *Teaching Evidence-Based Medicine,* which was held annually at NYAM. She recruited librarian tutors and librarian students for the program, developed the Evidence-Based Medicine Resource Center website as part of a three-year National Library of Medicine (NLM) Information Systems grant, led initiatives supporting the role of librarians in evidence-based medicine (EBM), including series of CE sessions, and convened an EBM special interest group at MLA annual conferences. In recognition of her tireless efforts to foster EBM training, she was made a Fellow of the New York Academy of Medicine in 2010.

Pat's personal interests were deep and varied: theater (she remained, after all, a sophisticated New Yorker, even after she left the city); Shakespeare (although she thought him unfair to Richard III); Star Trek (Kirk was her captain of choice); old movies (especially films starring Miss Barbara Stanwyck and the less-remembered Kay Francis); baseball (the Mets, never the Yankees); travel (although she had limited faith in the physics of airplanes); England (being of Irish descent, there were some limitations); tea (Earl Grey, hot, as per Captain Picard); and, of course, knitting. The annual Vogue Knitting Live conference and the Maryland Sheep & Wool Festival were as much fixtures in her calendar as the MLA national and chapter meetings. These interests would interact in strange and wonderful ways, sometimes in evidence at annual professional meetings. At one meeting in Madison, Wisconsin, in 2004, Pat went back and forth to Milwaukee to catch a Brewers game (eighty miles each way), after being assured (correctly) by a local MLA member that Milwaukee would be hard to miss—if you drove too far, you would fall into Lake Michigan. A meeting in Atlanta allowed her to the visit the *Gone with the Wind* museums (one part dedicated to the book, one to the movie). Pat hated inequality, but she admired the toughness of Scarlett O'Hara and was well aware that in real life, Margaret Mitchell subsidized local historically Black colleges and universities (HBCU) students. She had a keen awareness of how gender roles were represented in movies as opposed to “real life” and did a number of papers and presentations on how women doctors were portrayed by Hollywood.

In 2012, Pat was finally lured away from New York (marriage was part of the deal) and relocated to Maryland, where she took up a position at NLM's National Information Center on Health Services Research and Health Care Technology (NICHSR, now subsumed under NLM's Public Services Division). Although her duties and location were now very different, she maintained her MLA links at both the local and national level. In 2018, she became a Fellow of the Medical Library Association. She was also deeply involved with the merger of the New York/New Jersey and Philadelphia MLA chapters into the new Liberty Chapter. In one of its first official acts, the Liberty Chapter granted Pat a Lifetime Achievement Award in recognition of her decades of service.

Pat is survived by her husband, Stephen J. Greenberg, AHIP, and two stepchildren, Ilana Greenberg-Sud and Evan Greenberg, all of whom will miss her deeply.

